# Self-Regulated Learning Assessment in Young Soccer Players: Beyond Competitive Levels

**DOI:** 10.1519/JSC.0000000000004950

**Published:** 2024-09-20

**Authors:** Athos Trecroci, Luca Cavaggioni, Alessio Rossi, Tindaro Bongiovanni, Pietro Luigi Invernizzi, Damiano Formenti, Stefano Longo

**Affiliations:** 1 Department of Biomedical Sciences for Health,University of Milan, Milan, Milano, Italy;; 2 IRCCS Istituto Auxologico Italiano, Obesity Unit and Laboratory of Nutrition and Obesity Research, Department of Endocrine and Metabolic Diseases, Milan, Italy;; 3 Department of Research and Development, MyPowerSet, Rome, Italy;; 4 Performance Department, Palermo FC, Palermo, Italy; and; 5 Department of Biotechnology and Life Sciences, University of Insubria, Varese, Italy

**Keywords:** psychological skills, dribbling, change of direction, teamsport

## Abstract

Trecroci, A, Cavaggioni, L, Rossi, A, Bongiovanni, T, Invernizzi, PL, Formenti, D, and Longo, S. Self-regulated learning assessment in young soccer players: beyond competitive levels. *J Strength Cond Res* 39(1): e56–e61, 2025—This study explores self-regulated learning (SRL) among young soccer players, transcending the traditional focus on competitive levels. One hundred twenty-four soccer players of regional and provincial levels from under 14 to under 17 age groups voluntarily participated in the study and were combined into a single group. A median-split method based on total time performance was used to separate players into low performers (LPs) and high performers (HPs) from a 90° change of direction dribbling test. The self-regulation of learning—self-report scale for sport practice (SRL-SRS-SP) with a 5-factor solution (planning, reflection, effort, self-efficacy, and self-supervision) and 31 items was used. The score was based on a 1–5 Likert scale. The level of significance was set at *p*-value < 0.05. The SRL-SRS-SP total score by HP players differed significantly from that of LP players (*p* = 0.015). Regarding each subscale, HP players showed significantly higher scores than LP players (*p* = 0.011) for planning, whereas no differences were observed for the remaining factors (*p* ≥ 0.05). The main observation of this study revealed that HP soccer players had a greater level of engagement in the learning process with a remarkable ability to plan for specific improvement than LP peers. This suggests that the competitive level might not be the sole determinant of differences in SRL. Therefore, strength and conditioning coaches should emphasize metacognitive and motivational components because of their crucial role in enhancing technical and physical performance.

## Introduction

Self-regulated learning (SRL) is a psychological process indicative of an individual's ability to manage and refine motor skills by monitoring progress and adapting to different learning levels (13). According to Zimmerman (24), self-regulated learners are metacognitively, motivationally, and behaviorally proactive in their own learning process. This involves metacognition components such as planning, self-monitoring, evaluation, and reflection, with effort and self-efficacy contributing to the motivational aspect. These components play a crucial role in aiding athletes to recognize unwanted actions, understanding their negative impact on performance, and identifying effective actions that should be enhanced (15).

Soccer performance relies on physical, technical, tactical, and psychological aspects (3). The physical determinant ensures that the player can sustain explosive actions over time, performing effective dribbling, win duels, and engage in operative defensive and offensive maneuvers (1). However, attempts to improve soccer performance often focus on technical, tactical, and physical determinants and their interactions, sometimes overlooking metacognitive and motivational components of SRL that are supposed to directly influence an athlete's efficacy in each of the mentioned determinants (20). Indeed, an athlete's effort, which refers to the dedication put into their learning process, goes beyond the mere physical, technical, and tactical practice (15,17).

The metacognitive and motivational aspects of SRL are crucial in youth soccer. In the early stages, young players set goals, creating action plans for skill improvement (e.g., faster dribbling). They self-monitor during skill execution, engage in reflective practices, assess successes and shortcomings, and gauge proficiency (17). Setting a goal, creating a plan, self-monitoring, and reflection are processes that demand sustained optimal efforts, reinforcing belief in success with resilience to maximize performance, exemplified by goals like fast dribbling. Significantly, these SRL components emerge as pivotal discriminators in young soccer players' transition to professional ranks (5), potentially distinguishing success from nonsuccess (8). Indeed, a young individual who is more aware of his/her strengths and weaknesses (e.g., regarding technical skills) may be able to use this awareness to develop plans of action in an attempt to improve his/her skills (7). This would foster the ability to manage pressure related to adverse conditions, also helping to build a proactive mindset to succeed (6,7).

Previous studies delved into the psychological skills related to metacognitive and motivational SRL components among athletes of different competitive levels (2,4,9,11,15,17,18). Jonker et al. (9) noticed that an advanced sense of reflection distinguished between athletes at a high level of excellence and low-level peers in individual and team sports. This translates into a better ability of high-level athletes to constantly reflect on their own learning process and apply prior knowledge and strategies to future actions.

In the realm of soccer, heightened levels of reflection and effort have been associated with superior performance among young players (17). Conversely, in adult players, self-monitoring emerged as a predictor for inclusion in both elite and subelite groups, distinguishing them from those engaged at the recreational level (2). Furthermore, Reverberi et al. (15) observed that young elite soccer players demonstrated significantly elevated levels of planning, self-supervision, effort, reflection, and self-efficacy in comparison to their nonelite counterparts.

Determining the competitive status in sports, especially soccer, is variable and subjective, as noted in prior studies (10,21). Athletes may attain elite status one season and decline the next, underscoring the subjective nature of competitive ranking (21). Although prior research has emphasized psychological skills in categorizing performance, relying solely on competitive levels (2,4,9,11,15,17,18) may not provide a comprehensive understanding of the interplay between soccer performance and psychological skills. Conversely, technical skills offer a potentially stable, tangible, and accurate assessment criterion, serving as an objective indicator of players' performance alongside their proficiency.

It seems evident that SRL should be considered not only in relation to competitive level but also in close connection with technical skills, which are a fundamental aspect of soccer performance. This would be innovative and may reveal new insights into the interplay between sport-specific abilities and psychological skills in soccer.

Therefore, this study aims to explore SRL among young soccer players with different sport-specific abilities (i.e., dribbling), transcending the traditional focus on competitive levels. Based on the above considerations and the gaps in the current literature, we hypothesize significant differences in the level of SRL among young soccer players with different dribbling skill, regardless of their competitive ranking.

## Methods

### Experimental Approach to the Problem

This study used an observational design administered during a competitive season in subelite male academy soccer players. Players participated in the experimental procedure in May and were tested on an outdoor artificial turf at the same time of day (i.e., from 3 to 5 pm). Three sessions were planned. In the first session, height, sitting height, and body mass were measured with a stadiometer (SECA 213, Hamburg, Germany) and a portable scale (SECA 813, Hamburg, Germany) to the nearest 1.0 cm and 0.1 kg, respectively. In addition, the subjects were given a questionnaire on psychological skills to profile their SRL level. In the second session, they familiarized themselves with the sport-specific testing procedures. The third session occurred after 48 hours, in which all subjects underwent a 90° change of direction dribbling test and a 10-m straight-line sprint test to account for potential bias toward sprint capacity. Before performing the actual experimental evaluation, a standardized 5-minute warm-up was conducted based on forward and backward jogging, acceleration, deceleration, and jumps up to 5 m (19). All subjects were asked to refrain from ergogenic drinks 24 hours before the testing session.

### Subjects

From a priori power analysis, an observational design with a sample size up to 60 in each group (with a power of 0.9) detected a medium effect size (Cohen's *f* equal to 0.3) assuming a two-sided criterion that allows for a maximum type I error rate of α = 0.05. Accordingly, 124 soccer players of regional (*n* = 62) and provincial (*n* = 62) levels from under 14 to under 17 age groups were selected as volunteers to participate in the study. Instead of dividing the subjects into 2 groups based on their competitive ranking (i.e., regional and provincial levels), players were categorized as high performers (HP) or low performers (LP) based on the result of a soccer-specific dribbling task (see Table 1 for the subjects' characteristics). All subjects and their parents or guardians were informed about the purpose and potential experimental risks, and after a detailed description of the study, they provided written consent to participate in the investigation. The study was approved by the Ethical Committee of the University of Milan, in accordance with the Declaration of Helsinki.

**Table 1:** Subjects' characteristics including anthropometric variables and training status for both HP and LP groups.*

Ability level	Anthropometric variables			Training status	
	Age (range in years)	Height (m)	Sitting height (cm)	Body mass (kg)	Deliberate practice (h·wk^−1^)
HP	14–17	1.7 ± 0.1	89.2 ± 4.5	61.28 ± 9.5	7.18 ± 1.2
LP	14–17	1.7 ± 0.1	89.0 ± 4.7	61.30 ± 9.4	7.00 ± 1.0

* LP = low performer; HP = high performer.

### Procedures

#### Psychological Skill Assessment

Self-regulated learning was assessed by the Italian version of the Self-Regulation of Learning—Self-Report Scale (SRL-SRS-SP) by Bartulovic et al. (2), which was validated for youth soccer (15). This modified version of SRL-SRS-SP consists of 31 items wrapped in a 5-factor solution including the following subscales: planning (8 items, e.g., “*I develop a plan for resolving difficulties at practice*”); self-monitoring (8 items, e.g., “*While I am engaged in a practice task, I know how much of it I still have to complete*”); reflection (2 items, e.g., “*When facing difficulties at practice I* can *remain calm because I* can *rely on my coping abilities*”); effort (8 items, e.g., “*I put forth my best effort when performing tasks at practice*”); self-efficacy (5 items, e.g., “*No matter what comes my way at practice, I am usually able to handle it*”). Consistent with the original version (2), items 1–24 were evaluated using a 5-point Likert-type scale to gauge the frequency of specific occurrences during training, ranging from (1) “never” to (5) “always.” Meanwhile, items 25–31 were assessed on a Likert scale from 1 to 5 to measure the respondent's agreement with each statement, varying from “not at all” to “totally.” Total and subscale mean scores were computed for the analysis. For the SRL-SRS-SP administration, all subjects provided their responses in a quiet room with no interaction with other players in an attempt to keep the condition equal for all subjects.

#### Soccer-Specific Dribbling Assessment

The 90° change of direction dribbling test was arranged to assess each player's ability of sprinting over 10 m with a 90° turn while controlling the ball. This is a reliable test aimed to investigate the dribbling ability in youth soccer (19). All players were instructed to perform 3 trials in each direction (left and right) with 2 minutes of passive recovery in between. The distance between the starting line and the cone and between the cone and the finish line was set at 5 m each. The cone at the turning point had a square base (27 × 27 cm) with a 51 cm height. Excluding the initial ball touch and the ball touch for changing direction, subjects had to dribble the ball by at least 2 touches within the first (initial 5 m) and the second (final 5 m) paths. These instructions were provided to ensure that each player maintained constant control of the ball close to his foot, avoiding the need to chase after it, which would influence the dribbling assessment. Three Union of European Football Associations (UEFA) licensed experimenters observed the execution of each player, making sure the performance met the instructions. In case of noncompliance with the instructions and/or a collision/contact with the cone at the turning point, the players had to stop and repeat the trial after 2 minutes of recovery. An electronic timing system (WICHRO; Chronojump Boscosystem, Barcelona, Spain) was used to record the performance time. The gates were placed at 0.7 m of height, and the players’ departures occurred with their most advanced foot at 0.3 m behind the starting line (19). The best mean time between the right and left directions was used in the analysis.

#### Sprint Performance Assessment

A straight-line sprint test was executed over a 10-m distance. Each subject started at his own pace with the intent to accelerate maximally. Three trials interspersed with 2 minutes of rest were granted, with the best performance time included in the analysis. The instrument and setup used for recording time in the 90° change of direction dribbling test were also used in this evaluation.

### Statistical Analyses

Data normal distribution on the present sample size was verified by the Kolmogorov–Smirnov test (14). Absolute reliability was assessed using the coefficient of variation (CV) calculated as ([*SD*/mean] × 100). The intraclass correlation coefficient (ICC) with 95% confidence interval (95% CI) was calculated as a measure of relative reliability. In this study, the ICC was computed using a 2-way mixed model (3) for average measures (k) to assess the consistency in the performance tests. ICCs (3,k) were classified as *poor* (ICC < 0.5), *moderate* (0.5 ≤ ICC ≤ 0.75), *good* (0.76 ≤ ICC ≤ 0.90), and *excellent* (ICC > 0.90) (12). Confirmatory factor analysis (CFA) based on the adequate fit indices (root mean square error of approximation [RMSEA]; Comparative Fit Index [CFI]; Tucker–Lewis Index [TLI]; Standardized Root Mean Squared Residual [SRMR]) was used to assess the construct validity of the SRL-SRS-SP scale returned from the item scores. The median-split method was used to categorize HP and LP. The differences in the physical tests, SRL total, and subscales scores between HP and LP were tested using a univariate analysis of variance, with age and sprint performance as covariates. The effect sizes $\eta_{p}^{2}$ were calculated and interpreted as follows: $\eta_{p}^{2}$ = 0.01 indicates a small effect; $\eta_{p}^{2}$ = 0.06 indicates a medium effect; $\eta_{p}^{2}$ = 0.14 indicates a large effect. Pearson's correlation coefficient (*r*) was used to assess the relationships between SRL scores and dribbling performance. Statistical significance was set at *p* ≤ 0.05, with the analysis carried out by the Jamovi (16) software. Data were presented as mean ± *SD*.

## Results

**Figure 1.**
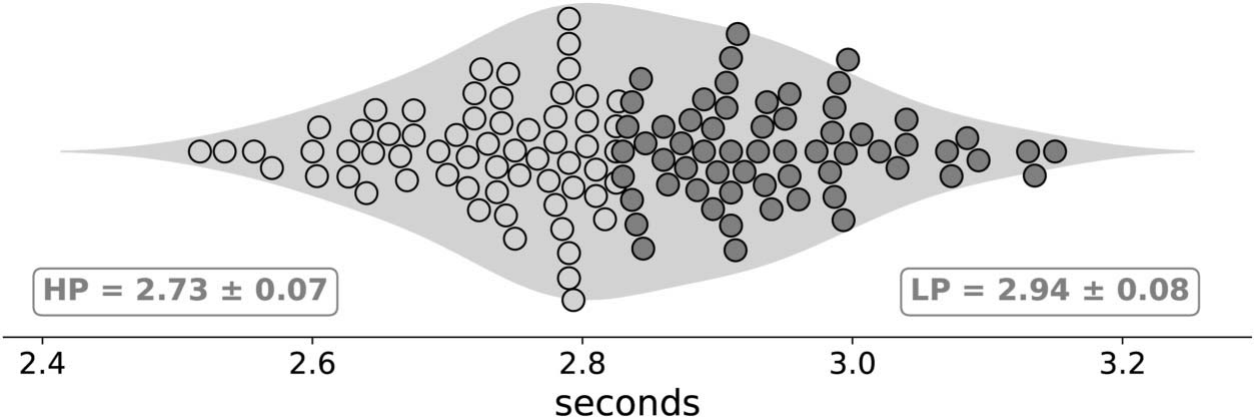
Violin plot of the 90° change of direction dribbling performance. The shaded area of the violin plot reveals the distribution of the performance time. The plot displays the distribution of times (mean ± *SD*) for high performers (HPs) on the left (light gray dots) and low performers (LPs) on the right (dark gray dots).

**Figure 2.**
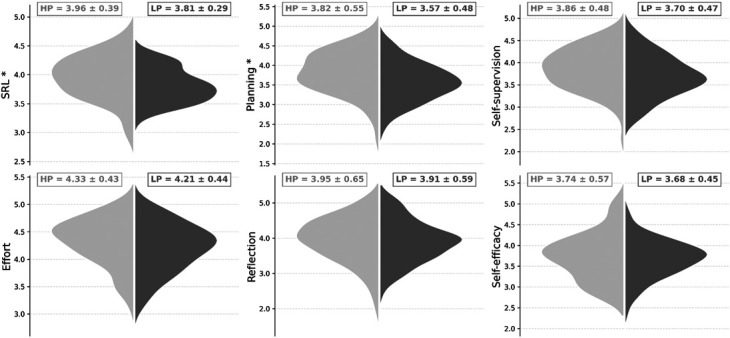
Violin plot of the self-regulated learning (SRL) total and subscores. The shaded area of the violin plots reveals the distribution of the SRL, planning, self-supervision, effort, reflection, and self-efficacy scores expressed in arbitrary units. The plot displays the distribution each SRL score (mean ± *SD*) for high performers (HPs) on the left (light gray dots) and low performers (LPs) on the right (dark gray dots). The asterisk indicates a significant (*p* < 0.05) difference between HPs and LPs.

In the 90° dribbling test, the right and left direction CV was 2.1 and 2.5%, respectively, with an *excellent* reliability for both directions (right direction: ICC = 0.92 [95% CI: 0.85–0.96]; left direction ICC = 0.90 [95% CI: 0.80–0.95]). In the sprint test, CV was 1.7% with an *excellent* reliability (ICC = 0.94 [95% CI: 0.87–0.98]). The adequate fit indices of CFA returned from the item scores were overall acceptable (χ^2^ = 590, df = 395, *p* < 0.001) with (RMSEA = 0.06 [0.05–0.07], CFI = 0.80, TLI = 0.77, SRMR = 0.085). From the best times coming from the 90° dribbling test (median = 2.83 seconds), 63 players were categorized as HP (≤2.83 seconds) and 61 players as LP (>2.83 seconds). The 90° dribbling best times of HP players were significantly lower than those of LP players (*F*_1,121_ = 222.4, *p* < 0.001) with a large effect ($\eta_{p}^{2}$ = 0.624; Figure 1). No significant differences (*F*_1,121_ = 2.19, *p* = 0.141, $\eta_{p}^{2}$ = 0.018) were observed in the sprint performance between HP (1.77 ± 0.07 seconds) and LP (1.79 ± 0.07 seconds). The SRL-SRS-SP total score of HP players differed significantly from that of LP players (*F*_1,120_ = 6.13, *p* = 0.015) with a small effect ($\eta_{p}^{2}$ = 0.049; Figure 2A). Regarding each subscale, HP players showed significantly higher scores than LP players (*F*_1,121_ = 6.71, *p* = 0.011) for planning (Figure 2B), with a small effect ($\eta_{p}^{2}$ = 0.053). No significant differences were observed for self-supervision (*F*_1,121_ = 3.83, *p* = 0.052, $\eta_{p}^{2}$ = 0.031; Figure 2C), effort (*F*_1,121_ = 3.10, *p* = 0.081, $\eta_{p}^{2}$ = 0.025; Figure 2D), reflection (*F*_1,121_ = 0.33, *p* = 0.562, $\eta_{p}^{2}$ = 0.003; Figure 2E), and self-efficacy (*F*_1,121_ = 0.33, *p* = 0.563, $\eta_{p}^{2}$ = 0.003; Figure 2F). Significant negative relationships were found between SRL score and dribbling performance (*r* = −0.18; *p* = 0.04) and between planning score and dribbling performance (*r* = −0.21; *p* = 0.001).

## Discussion

The primary observation of this study reveals that HP soccer players exhibit a greater level of engagement in the learning process compared with their LP peers, coupled with a remarkable ability to plan for specific improvement, irrespective of their level of play. This underscores the crucial role of soccer-specific performance in discerning SRL among young soccer players, suggesting that proficiency in dribbling skills may contribute to the development of SRL in youth soccer. Our initial hypothesis concerning the differences in SRL among young soccer players contingent upon their dribbling skills was substantiated.

To the authors' present knowledge, this study represents the first attempt to observe an existing interplay between sport-specific skills, specifically dribbling, and the psychological aspects of SRL, with a particular emphasis on the planning component. Planning is the key cognitive process of goal-oriented approaches to practicing tasks, deciding on approaches to a task, and task analysis (22). It was previously observed as a part of the main metacognitive processes in highly self-regulated adolescent soccer players (18) who possibly gain knowledge and skills with increased effectiveness (24). This holds especially true when it comes to acquiring technical competence.

In this study, players exhibiting superior dribbling skills demonstrated higher planning scores compared with their less skilled counterparts. It may be inferred that they exhibited a heightened ability to exert control over the progression of their technical performance. This is even more apparent when examining the comparable deliberate practice time between HP and LP players in Table 1. Toering et al. (17) found reflective ability to differentiate between performance levels against an equal total number of practice hours, suggesting that higher self-regulated soccer players (i.e., elite athletes) make the most of practice than lower self-regulated peers (i.e., nonelite players) by putting effort in task execution and reflecting more upon their performance. High performers may have benefited more from training practice than LPs by enhancing their dribbling skills through a strategic and specific planning process. For instance, HPs may have planned to improve their dribbling skills by adopting precise ball touches, short and frequent strides, and low center of mass (23) instead of dwelling on a general idea to refine ball control technique in a time-constraint condition. This might be reasonably assumed even though no information on complete players' practice history (combination of deliberate practice and deliberate play) was available. Notably, a negative correlation between SRL score and dribbling performance was observed, particularly with the planning subscale. This supports the potential link between the metacognitive aspect of SRL and technical skills (i.e., dribbling). By contrast, although SRL and dribbling skills appear related, changes in one variable do not necessarily entail changes in the other. Moreover, the relationship appears weak, and further research is needed to explore the causal relationships and understand the underlying mechanisms that connect these variables.

In soccer, most studies focusing on SRL and its metacognitive and motivational components showed differences in the processes of reflection, effort, and self-monitoring according to the competitive level (2,9,17). Previously, it was found that young elite players invest much more effort in performing physical tasks and reflect more on their previous actions (what went right or what went wrong) than nonelite ones (17). Similarly, Jonker et al. (9) observed that reflection was the main discriminator in both individual and team sports in young adolescent athletes. Conversely, self-supervision was the most important characteristic discriminating between competitive levels in adult soccer players (2). An interesting side finding of this work was that self-supervision may deserve careful attention because the difference between HP and LP was close to reach conventional statistical significance (*p* = 0.055). The actual nonsignificant *p*-value does not preclude the presence of interesting trends and potential links with dribbling skills. At most, it may hint at a subtle inclination among HP players to better supervise all aspects of their dribbling execution (also checking for successes and shortcomings while doing it) unlike LP peers. Further studies with larger sample sizes are needed to verify these hypotheses.

Overall, besides planning, the lack of differences in the rest of SRL processes might depend on the proper choice of assessing a player's technical performance (objective assessment) versus competitive ranking (subjective assessment). In fact, players' competitive ranking may fluctuate unexpectedly (e.g., due to events like injuries) and subjectively (e.g., influenced by a coach's perspective) compared with the stability and consistency of their technical ability (such as actual dribbling proficiency). Information on SRL obtained only from competitive ranking could limit the understanding of the process of talent identification and development in soccer. The change in competitive status could alter the context (support and resources) and surrounding expectations (coach's expectations creating more anxiety), influencing the subjective perception of players' SRL abilities. Collectively, these findings indicate that distinct psychological processes may emerge when evaluating SRL concerning competitive level versus technical proficiency. This insight is crucial for coaching staff, particularly in the context of talent identification within soccer (21). However, existing literature lacks comprehensive data supporting this specific area of investigation. The pivotal decision between objectively assessing technical performance and relying on subjective competitive rankings remains an interesting area to be explored concerning the evaluation of psychological skills, albeit beyond this study's scope. Further research is required to validate the effectiveness of assessing SRL abilities based on sport-specific skills and ascertain its potential superiority over using competitive rankings as an evaluation metric.

Although this study offers novel findings on the interplay between SRL and dribbling skill, it remains observational in nature, not an intervention, suggesting a need for a more cautious interpretation of the findings. Moreover, it is important to recognize certain limitations that require acknowledgment. Although the sample size was large enough to detect a smaller effect originating from a priori analysis, a bigger sample would have helped to minimize the risk of type II error (perhaps by offsetting values of power >0.9) having less than 10% chance of failing to detect an effect that exists, such as for self-supervision. Unfortunately, there were no previous data to take as a reliable basis. The SRL-SRS-SP scale, though widely familiar to subjects because of its frequent use during the season, lacked established test–retest reliability, and a larger sample size would have also strengthened its internal validity. Assessing dribbling performance in a “closed” condition provided valuable insights. However, a more comprehensive understanding of the interplay between technical and SRL skills could be gained by evaluating performance in a more complex and ecological “open” condition, like small or big-sided games.

In summary, our study provides valuable insights into the intricate interplay between sport-specific skills, particularly dribbling, and the psychological components of SRL, with a specific focus on the planning process. The findings emphasize the pivotal role of planning, a key cognitive process related to goal-oriented practice, task decision-making, and task analysis, in discerning the ability to self-regulate the learning process among young soccer players.

**Practical Applications**

Understanding the influence of sport-specific skills on the self-regulation of learning can offer valuable insights for devising targeted and individualized training strategies. This understanding may shed light on psychological skills that could be cultivated further. For instance, if mastering a particular technical skill (such as quick ball dribbling) demands substantial goal-oriented practice, task decision-making, and task analysis, training programs could incorporate methodologies aimed at enhancing these specific psychological attributes. Consequently, coaching staff are encouraged to adopt a more comprehensive approach to assessing soccer performance, where psychological determinants synergistically intertwine not only with technical aspects but also with tactical and physical elements.
